# Segment-Wise Genome-Wide Association Analysis Identifies a Candidate Region Associated with Schizophrenia in Three Independent Samples

**DOI:** 10.1371/journal.pone.0038828

**Published:** 2012-06-18

**Authors:** Thomas E. Gladwin, Eske M. Derks, Genetic Risk and Outcome of Psychosis (GROUP), Marcella Rietschel, Manuel Mattheisen, René Breuer, Thomas G. Schulze, Markus M. Nöthen, Douglas Levinson, Jianxin Shi, Pablo V. Gejman, Sven Cichon, Roel A. Ophoff

**Affiliations:** 1 Department of Psychiatry, Rudolf Magnus Institute of Neuroscience, University Medical Center Utrecht, Utrecht, The Netherlands; 2 Division of Genetic Epidemiology in Psychiatry, Central Institute of Mental Health, Mannheim, Germany; 3 Institute of Human Genetics, University of Bonn, Bonn, Germany; 4 Institute for Medical Biometry, Informatics and Epidemiology, University of Bonn, Bonn, Germany; 5 Department of Genomics, Life and Brain Center, University of Bonn, Bonn, Germany; 6 Department of Psychiatry and Psychotherapy, University Medical Center, Georg-August Universität, Göttingen, Germany; 7 Department of Psychiatry, Stanford University School of Medicine, Palo Alto, California, United States of America; 8 National Cancer Institute, Bethesda, Maryland, United States of America; 9 Department of Psychiatry and Behavioral Sciences, Northshore University Health System and University of Chicago, Evanston, Illinois, United States of America; 10 University California Los Angeles, Center for Neurobehavioral Genetics, Semel Institute for Neuroscience and Human Behavior, Los Angeles, California, United States of America; 11 ADAPT Lab, Department of Developmental Psychology, University of Amsterdam, Amsterdam, The Netherlands; 12 Department of Psychiatry, Academic Medical Center, University of Amsterdam, Amsterdam, The Netherlands; Johns Hopkins University, United States of America

## Abstract

Recent studies suggest that variation in complex disorders (e.g., schizophrenia) is explained by a large number of genetic variants with small effect size (Odds Ratio∼1.05–1.1). The statistical power to detect these genetic variants in Genome Wide Association (GWA) studies with large numbers of cases and controls (∼15,000) is still low. As it will be difficult to further increase sample size, we decided to explore an alternative method for analyzing GWA data in a study of schizophrenia, dramatically reducing the number of statistical tests. The underlying hypothesis was that at least some of the genetic variants related to a common outcome are collocated in segments of chromosomes at a wider scale than single genes. Our approach was therefore to study the association between relatively large segments of DNA and disease status. An association test was performed for each SNP and the number of nominally significant tests in a segment was counted. We then performed a permutation-based binomial test to determine whether this region contained significantly more nominally significant SNPs than expected under the null hypothesis of no association, taking linkage into account. Genome Wide Association data of three independent schizophrenia case/control cohorts with European ancestry (Dutch, German, and US) using segments of DNA with variable length (2 to 32 Mbp) was analyzed. Using this approach we identified a region at chromosome 5q23.3-q31.3 (128–160 Mbp) that was significantly enriched with nominally associated SNPs in three independent case-control samples. We conclude that considering relatively wide segments of chromosomes may reveal reliable relationships between the genome and schizophrenia, suggesting novel methodological possibilities as well as raising theoretical questions.

## Introduction

The statistical power to detect genetic effects in Genome Wide Association (GWA) studies of complex disorders is hindered by multiple testing problems and small effect sizes of single SNPs [1]. Alternative methods for analyzing GWA data which consider larger scale relationships between phenotype and SNP or single genes may help address these problems, and may have biological implications concerning the organization of genetic information. The aim of the current study was to determine whether relatively broad segments of the genome, as opposed to specific SNPs, could be related to schizophrenia. Our approach was to test per segment whether significantly higher numbers of nominally related SNPs were present than expected based on chance.

Schizophrenia is a complex psychiatric disorder which is to a large extent influenced by genetic effects [2]. Recently, based on the findings of Genome Wide Association (GWA) studies it has been suggested that about 30% of the genetic variation is explained by a large number of SNPs with small effect sizes [1]. The statistical power to detect the effect of single SNPs is low which may explain the missing heritability for schizophrenia [3]. In an effort to deal with this statistical problem, Moskvina and colleagues [4] used a gene-based approach to perform a GWA by determining the excess of nominally significant (P<.05; P<.01, and P<.001) disease associated SNPs within genes, observing that significantly more SNPs within genes showed evidence for association with schizophrenia than expected by chance. Although these results are important, a limitation of the method is the a priori exclusion of the genome outside the genic regions. Further, only segments at the scale of single genes were considered. True association signals may also be located outside the boundaries of genes; for example, studies have shown the existence of long-range regulatory elements which suggest that the effects of functional gene domains may extend far beyond their transcription unit [5].

In this study, we will study the excess of nominally significant SNPs within large segments (2–32 Mbp) of the genome. This approach uses all genome-wide genotype data, searching for possible clusters in the distribution of disease-related variation over the genome without prior restrictions to gene locations. The rationale of this approach is the premise that genes contributing to schizophrenia may not be randomly distributed across the genome but may be clustered in coordinated expression domains [6] or chromosomal territories [7]. Woo and colleagues have examined microarray data collected in mice and humans and investigated whether genes showing coexpression (i.e., genes with comparable expression profiles) are clustered in the genome. They have detected strong and statistically significant enrichment of coexpression among pairs of genes whose distances fall within the sub-megabase range. In addition, they report a weaker but still significant enrichment of coexpression among genes with distances spanning tens of megabases. Lieberman-Aiden and colleagues used Hi-C to study long-range interactions between specific pairs of loci [8]. In Hi-C, cells are fixed with formaldehyde, causing interacting loci to be bound to one another by means of covalent DNA-protein cross-links [9]. Lieberman-Aiden showed that the probability of physical contact decreases as a function of genomic distance on chromosome 1. We therefore developed the idea that chromosomal segments potentially represent meaningful entities in the sense that SNPs within a particular segment have a higher chance to be involved in the same biological or functional pathway compared to SNPs from different segments. We varied the segment sizes from 2 Mbp to 32 Mbp as we wanted to explore a wide variety of segment sizes and these sizes appear to be reasonable based on the results of the studies on genetic coexpression.

The aim of this paper is to present a novel, data-driven approach for the analysis of GWA data based on the possibility that subsets of weakly associated SNPs are located within relatively broad segments of DNA, spanning both genic and intergenic locations. Tests performed on three independent data sets showed that a relatively broad region of chromosome 5 is significantly associated with schizophrenia. The result provides novel information on the genetic basis of schizophrenia and supports the assumption that at least part of the genetic variation underlying schizophrenia involves genetic information contained within contiguous segments of the genome.

## Results

**Figure 1:**
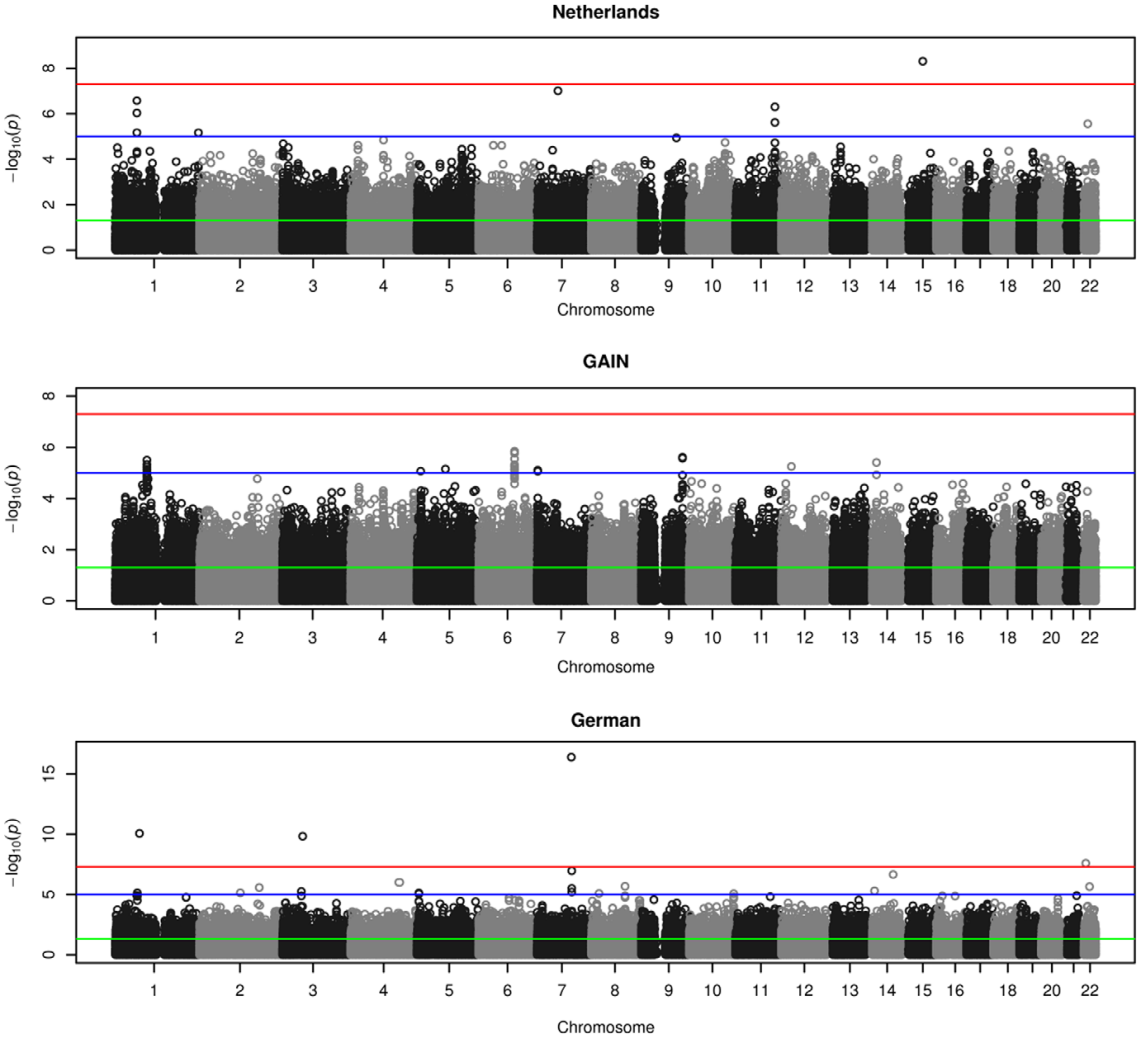
“Manhattan plot of the 22 autosomal chromosomes”. This figure shows at the y–axis the p-values of the SNPs in a GWA analysis. The chromosomes are shown at the x-axis. The red line indicates a p-value of 10-7, the blue line indicates a p-value of 10-5 and the green line indicates a p-value of .05.

**Figure 2:**
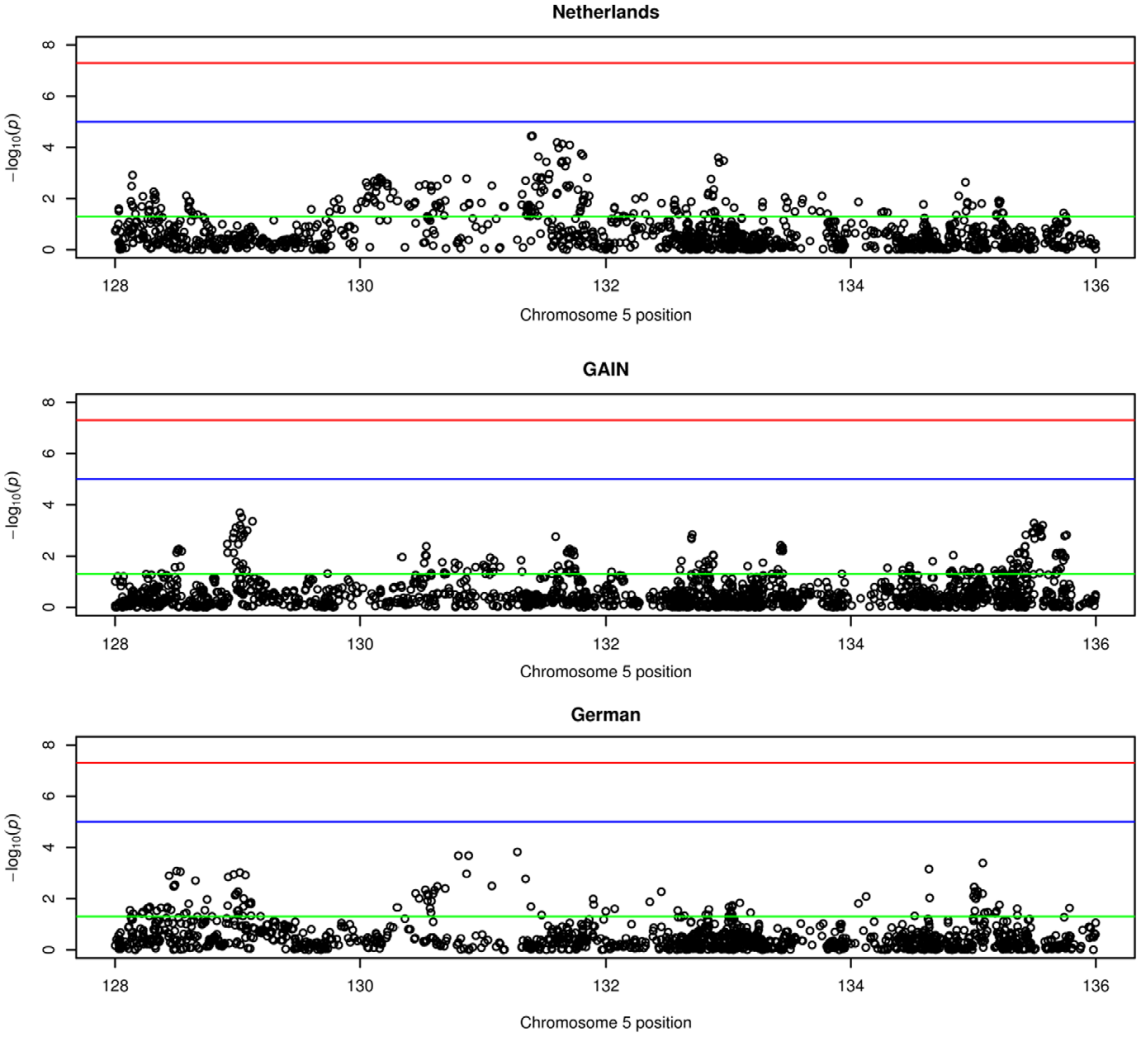
“Manhattan plot of the top segment located at chromosome 5 (128–136 **Mbp)”.** This figure shows at the y–axis the p-values of the SNPs located at chromosome 5 (128–136 Mbp). The chromosomes are shown at the x-axis. The red line indicates a p-value of 10-7, the blue line indicates a p-value of 10-5 and the green line indicates a p-value of .05.

One region on chromosome 5q was found to be associated with schizophrenia in all three samples, for all segments widths above 2 Mbp: chromosome 5q23.3-q31.3 (128–136 Mbp). Different widths showed only small variations in the borders of the region: width 4 Mbp, 132–136 Mbp; width 8 Mbp, 128–136 Mbp; width 16 Mbp, 120–136 Mbp; width 32 Mbp, 128–160 Mbp. When testing the significance of the regions using Fisher’s combined probability test for combining *p*-values, the segments for the 16 and 32 Mbp width survived stringent Bonferroni correction. In the 5q23.3-q31.3 (128–136 Mbp) region, the number of observed nominally significantly SNPs was higher than the expected number in the sample from the Netherlands (N observed = 216; N expected = 66), the GAIN sample (N observed = 189; N expected = 95), and the German sample (N observed = 119; N expected = 66). As described in the methods section, the significance in each cohort was determined based on permutation testing, to account for possible linkage disequilibrium in this region. The results of these tests are summarized in Table 1. Manhattan plots of the 22 autosomal chromosomes and the significantly associated region at chromosome 5 are shown in Figures 1 and 2, respectively.

**Table 1:** Metasignificance of segments located in chromosome 5 (128–160 Mbp).

Segment width [Mbp]	Replicable region [Mbp]	p (Netherlands)	p (GAIN)	p (Germany)	Fisher’s combined probability test
4	chr5: 132–136	0.017	0.009	0.025	3.49 E-4
8	chr5: 128–136	0.001	0.008	0.026	2.80 E-5
16	chr5: 120–136	0.001	0.016	0.013	2.80 E-5
16	chr5: 128–144	0.001	0.021	0.001	3.67 E-6
32	chr5: 128–160	0.001	0.023	0.001	3.98 E-6

*Note*. Segment width refers to the width of regions over which tests of the number of nominally significant SNPs were tested. The replicable region indicates the location of the segment. The p-values provide the results of permutation based tests.

The region was found to contain various (disease-related) genes, as shown in Table 2. Table 2 includes information on the genes in that region that have previously reported to be associated with disease as denoted in the UCSC Genome Bioinformatics site (NCBI36/hg18) (http://genome.ucsc.edu/). Furthermore, we included information on the genes that were previously found to be associated with schizophrenia; this information was obtained from the Schizophrenia Research Forum (www.schizophreniaresearchforum.org). Finally, of all disease-related genes reported in the UCSC Genome Bioinformatics site, we denoted the phenotypes with which these genes have been associated.

**Table 2:** Overview of disease associated genes located within the significantly associated region at chromosome 5 (128–136 Mbp).

Start position(Mbp)	End position (Mbp)	Gene name	Database	Phenotype associations	Empirical p set-based test (Netherlands)	Empirical p set-based test (GAIN)	Empirical p set-based test (German)
130,627,600	130,758,281	*CDC42SE2*	GAD; SZGENE	Immune (asthma IgE)	.038	.077	.014
130,789,503	130,998,828	*RAPGEF6*	SZGENE		.025	.124	.001
131,170,738	131,375,769	*ACSL6*	GAD; SZGENE	Psychiatric (schizophrenia)	.069	.097	.001
131,424,245	131,426,795	*IL3*	GAD; SZGENE	Immune (asthma; atopy, rheumatoid arthritis)	.005	1	1
131,437,383	131,439,758	*CSF2*	GAD	Immune (asthma; atopy; dermatitis and eczema, atopic dermatitis, dermatitis and eczema)	.020	1	1
131,621,285	131,637,046	*RIL*	GAD	Metabolic (bone density)	.001	.094	1
131,658,043	131,707,798	*SLC22A4*	GAD	Immune (Crohn’s disease, Crohn’s disease ulcerative colitis, rheumatoid arthritis)	.002	.070	1
131,733,342	131,759,202	*SLC22A5*	GAD	Immune (Crohn’s disease, Crohn’s disease ulcerative colitis)	1	.027	1
131,846,683	131,859,158	*IRF1*	GAD	Immune, Infection (asthma, hepatitis C, hepatitis C, chronic)	.003	1	1
131,905,034	131,920,427	*IL5*	GAD	Immune (asthma atopy, dermatitis and eczema)	1	1	1
132,021,763	132,024,700	*IL13*	GAD	Immune, Infection, Other, Pharmacogenomic, Renal (multiple phenotypes)	1	1	1
132,037,271	132,046,267	*IL4*	GAD; SZGENE	Cardiovascular, Hematological, Immune, Infection, Other, Renal, Reproduction (multiple phenotypes)	1	.091	1
132,224,776	132,228,376	*GDF9*	GAD	Reproduction (premature ovarian failure)	.041	1	1
132,415,560	132,468,608	*HSPA4*	GAD	Reproduction (preterm delivery)	.053	1	.018
133,478,300	133,511,819	*TCF7*	GAD	Immune (diabetes, type 1)	.049	1	1
134,807,802	134,810,937	*C5orf20*	GAD	Immune (asthma IgE)	1	.119	1
134,897,870	134,899,538	*NEUROG1*	GAD; SZGENE	Psychiatric (schizophrenia)	.014	1	1
135,255,833	135,259,415	*IL9*	GAD; SZGENE	Immune (asthma)	1	1	1
135,300,162	135,305,266	*FBXL21*	SZGENE		1	.047	1
135,392,596	135,427,406	*TGFBI*	GAD	Other (BIGH3 gene mutations, granular corneal dystrophy, Lattice corneal dystrophy type 1)	1	.007	1

Note: The table shows genes that have previously reported to be associated with disease based on the UCSC Genome Bioinformatics site (NCBI36/hg18) (http://genome.ucsc.edu/) and genes previously found to be associated with schizophrenia based on the Schizophrenia Research Forum (www.schizophreniaresearchforum.org). The final column represents the phenotype and disease associations according to the UCSC Genome Bioinformatics site.

Set-based tests were performed in Plink to assess the association between SNPs within a particular gene and case-control status. This test uses permutation to determine the significance. The default values were used (r-squared = .5; p-value = .05; maximum number of SNPs within a gene = 5); more details can be found at http://pngu.mgh.harvard.edu/~purcell/plink.

Follow-up analyses were performed for the chromosome 5 (128–136 Mbp) segment width to test the sensitivity of the results to the parameters of the analysis (Table 3). In the original analyses, the threshold for nominal significance was .05. The analysis was repeated with different values for the nominal criterion level per SNP. For p = 0.01, a trend was found for the same region on chromosome 5,. For p = 0.1, the region remained significantly associated with schizophrenia status. Second, the original analysis (nominal p = .05) was repeated following EIGENSTRAT [10] correction, after which the region remained significant in two samples (p = 0.004, p = 0.001), and was close to the cut-off of .0264 in the remaining sample (p = 0.04). Again, the Fisher’s combined probability tests show highly similar results with p-values below the cut-off of 1.8 E-5 for nominal significance levels of .05 and .1 but not for p = .01. For an explanation of the rationale for the chosen cut-off values of .0264 and 1.8 E-5, please see the methods section.

**Table 3:** Results of the sensitivity analyses: a comparison of different nominal p-values.

Nominal p-value	p (Netherlands)	p (GAIN)	p (Germany)	Fisher’s combined probability test
.05	0.001	0.023	0.001	3.98 E-6
0.01	0.002	0.04	0.06	4.2 E-4
0.1	0.001	0.001	0.003	6.4 E-7
EIGENSTRAT	0.004	0.001	0.04	2.23 E-5

*Note*. Results for variations of the method for the 32 Mbp width region on chromosome 5, 128–160 bp. Nom 0.01: the analysis was performed using p = 0.01 as the cutoff for nominal SNP-wise significance. Nom 0.11: the analysis was performed using p = 0.1 as the cutoff for nominal SNP-wise significance. EIGENSTRAT: the analysis was performed on data corrected for population stratification using the EIGENSTRAT procedure.

## Discussion

The segment-wise method decreases the number of statistical tests from around 500,000 SNPs to the order of hundreds or thousands of segments. The fact that broad regions of 4 to 32 Mbp widths (but not of 2 Mbp) were found to contain increased numbers of nominally significant associated single SNPs suggests that the combination of information over larger segments can increase the strength of the segment-wise association signal. Clearly, the current approach is only appropriate for hypotheses or methods aimed at regionally clustered genetic information. Further, the increased power to detect association using large segments of the genome comes at the cost of a reduction in regional specificity. Based on our results, we do not know which specific genetic polymorphisms are involved with schizophrenia. The current method could therefore be seen as a first-sweep method which identifies regions of interest which should then be studied more extensively, but it may also indicate a distribution of schizophrenia-related genetic information that involves clusters over the genome.

There are at least three possible explanations for a finding of apparently clustered genetic information, measured via the combined effect of a large number of neighbouring SNPs. First, it is possible that the positive association arises due to multiple schizophrenia associated genes which are located in the same segment. A number of previously reported candidate genes for schizophrenia are indeed located in the chromosome 5 region which was highlighted in this study. Second, a more speculative but intriguing possibility is that associations between disease and the genome may exist on a different level of resolution; i.e., information may be present in the higher-order structure of the genome [11]; as well as in single genes. The higher-order organization of the genome comprises the folding of the DNA into chromatin fibers, chromosome domains, and ultimately, chromosomes and has been shown to be functionally important for gene regulation and control of gene expression programs [12]. The spatial organization of the genome may facilitate the communication between genomic elements by bringing the regulatory elements in close spatial proximity [11]. We could therefore hypothesize that it is advantageous for genes which are related to the same biological pathway to be physically close (i.e., in the same chromosome domain) since that could potentially facilitate the regulation of the co-expression of these genes. A third possibility is that the chromosome 5q region includes a series of independent, segregating indels that are in LD with the SNPs in this region. This would imply that the rare genetic variants result in synthetic associations between case-control status and the common SNPs [13].

The fact that we have found regional clustering of association signal at chromosome 5q (128–136 Mbp) suggests that a relatively large proportion of the genes in this segment plays a role in schizophrenia. Whether the genes in this segment are involved in the same biological or functional pathway should be investigated in future studies. A further question for future research is whether our approach could be relevant for other psychiatric or non-psychiatric complex traits. If the clustering of association signal is indeed explained by the higher-order organization of the genome, we might expect similar findings for other complex traits in which many genes within multiple biological pathways are involved. Colocalization of genes might be advantageous for other complex phenotypes for which the coexpression of many different genes has to be regulated. It would therefore be interesting to apply this approach to other complex phenotypes, such as height. In addition, we could investigate the role of the 5q region in, for example, bipolar disorder which is partly influenced by the same genetic risk factors as schizophrenia [1].

Genetic variants in the 5q region have previously been found to be associated with schizophrenia (e.g., *IL3, IL4, IL9, NEUROG1*). In addition, it is striking that many of the remaining genes are reported to be associated with immune diseases, including asthma, Crohn’s disease and type-I diabetes. The prevalence of asthma [14] and diabetes has been reported to be increased in patients with schizophrenia. The association with diabetes is not merely due to disease-related factors as the prevalence of diabetes is also increased in the relatives of schizophrenia patients and familial (e.g., genetic) factors may play a role [15]. Furthermore, there is increasing evidence of immune involvement in schizophrenia [16]. Therefore, the finding of many immune-related genes in the 5q region seems to further substantiate the segment-wise approach. Regarding previous work, we note that the method used in the current study may complement previous GWA studies. A large number of weakly associated SNPs could result in a highly significant segment-wise association while each individual SNP would fail to survive multiple testing correction. Conversely, a single highly significant SNP, with a sufficiently low p-value to survive Bonferroni correction, would be insufficient to highlight the whole segment containing it.

Recently, the findings of three multicenter studies were reported [1], [17], [18]. In the study of Stefansson and colleagues, a variant upstream of *neurogranin* (*NRGN*; *p* = 2.4×10^−9^) reached genome-wide significance. A meta-analysis of the three GWA studies (8,008 cases and 19,077 controls) identified a cluster of genome-wide significant SNPs in substantial LD in the MHC region on chromosome 6p22.1 [1], [17], [18]; this region was not consistently replicated in our segment-wise analyses. Although no segment reached our cut-off for statistical significance, when we specifically tested this region (chromosome 6: 26–28 Mb), we did find an increased number of nominally significant SNPs in each sample (p = 0.049 for Utrecht, p = 0.03 for GAIN, p = 0.009 for German; Fisher’s combined probability test p = 0.001). Beyond the methodological points mentioned above, the lack of statistical significance may be explained by the fact that, although the MHC region is quite large, spanning almost 5 Mb, the five genome-wide significant markers cover <2 cM [18]. It is possible that correction for linkage disequilibrium with permutation-based significance testing reduced the power to detect such relationships.

In conclusion, we found an association between schizophrenia and a large (8 Mbp) chromosomal region, spanning multiple genes. The segment-wise method proposed here complements a single SNP analysis as it increases the chance of detecting association which is clustered in a region but does not detect association if it is actually limited to one or few single SNPs. Further studies (e.g., based on the analysis of patterns of gene expression) are needed to gain more knowledge on the mechanisms which explain the clustering of genetic information in large segments of the genome.

## Materials and Methods

### Study Samples

All three schizophrenia GWA data sets have been described before. Briefly, the first set consists of 728 schizophrenia patients and 653 healthy controls from The Netherlands genotyped with the Illumina HumanHap550 BeadArrays [18]. The second sample includes 1,172 patients diagnosed with schizophrenia and 1,378 healthy controls of European descent from Molecular Genetics of Schizophrenia (MGS) dataset phase I for which genotypes were provided by the Genetic Association Information Network (GAIN) from the Affymetrix 6.0 array [17]. The third sample comprised 485 schizophrenia patients (described in Stefansson et al. [18]) and 1,363 population-based controls (described in Treutlein et al. [19]) from Germany genotyped with Illumina HumanHap550 BeadArrays.

### Ethics Statement

The studies were approved by the standing ethics committee, and all subjects gave written informed consent in accordance with the committee’s guidelines.

### Statistical Analysis

All chromosomes from one to 22 were divided into segments of varying width (see below), overlapping by 50%. For each SNP in the segment an adjusted Armitage trend test was performed (i.e., a test of the correlation of the number of rare alleles in the genotype and the phenotype). For each segment, it was tested whether the number of nominally (p<.05) significant SNPs in the segment was higher than expected by chance. Linkage disequilibrium was accounted for by using permutation testing, in which case-control status was randomized using 1000 permutations. In this way, each segment was associated with a *p*-value for the number of nominally significant associated SNPs it contained: this *p*-value was termed the metasignificance. The method was explored using a range of segment widths: 2, 4, 8, 16, and 32 Mbp. The significance level was corrected for the number of tests in the worst-case scenario, i.e., the number of tests when using segments of 2 Mbp width. Thus the probability of a false positive was at least Bonferroni-controlled at 0.05 for each segment width, and analyses involving different segments widths (and hence a different number of metasignificance tests) did not differ in terms of statistical threshold. The worst-case number of tests was 2729 which required a Bonferroni corrected *p*-value of 1.8 E-5. A segment was considered significant if the *p*-value was below the nominal criterion of *p* = 0.0264 in all three samples, so that the chance of a false positive occurring in all three independent samples was the required 0.0264^Λ^3 = 1.8 E-5. Note that tests were one-sided: only an increased number of SNPs in a segment would be considered a significant result for a sample, regardless of the *p*-value. This allows the probability of a compound event of a segment being significant at 0.0264 in all three independent samples to be calculated as the 0.0264^Λ^3 = 1.8 E-5. Analyses were performed using custom software in Matlab (The MathWorks, Inc, 1984).

We acknowledge that statistical power using this approach will depend on a number of factors, including the power of the individual SNP tests, the number of participants, the number of SNPs measured in each segment, and the correction for multiple testing. In the current study, the choice was made to be reasonably strict in terms of correction; in future work, the statistical power could be increased by considering only broader segments which would decrease the number of tests. Since we aimed to thoroughly explore the sensitivity of our method to the specific segments sizes used, we used a strict correction for the total number of tests at segment size 2 Mbp. We did not correct for the total number of segment sizes tested since the overlap between segment sizes would be considered to be problematic and the tests of different segment sizes in the same region can be expected to be highly correlated. Correction for the total number of segment sizes would therefore result in tests which are overly conservative.

Further, the permutation procedure introduces noise in the results, which could present a problem for results that are close to the threshold for sample-wise significance. To address this, we also calculated Fisher’s combined probability test for the combination of p-values over the sample. This method is less sensitive to thresholding and may be more robust, but tests a somewhat different null hypothesis than the compound-significance test of replicability. We note that when testing all segments using Fisher’s combined probability test, highly similar results to the compound-significance test were found. No additional regions were found that could have been undetected due to power problems with the compound event approach. Furthermore, for the 16 and 32 Mbp segment widths, *p*-values were lower such that the tests survived Bonferroni correction for all tested segments summed over all segment widths.

In follow-up analyses focussed on the 32 Mbp segment, potential artefacts due to population stratification were accounted for by adjusting case/control status for the first 10 EIGENSTRAT components [10]. Logistic regression was used with the binary variable encoding case– control status as a dependent variable and the EIGENSTRAT components as independent variables. The residual scores were used in subsequent statistical analyses. Further, the analyses were repeated for different nominal significance cut-offs per SNP, in order to determine the robustness of the method to this parameter.

The UCSC Genome Bioinformatics site (http://genome.ucsc.edu/) was used to find genes previously found to be associated with disease in the region. The March 2006 (NCBI36/hg18) was used to identify Phenotype and Disease Associations using the GAD view. Furthermore, we have used the Schizophrenia Research Forum (www.schizophreniaresearchforum.org) [20] to obtain information on genes that have previously reported to be associated with schizophrenia. Searches were performed at October 6, 2011.
